# Lab-on-Chip Microsystems for *Ex Vivo* Network of Neurons Studies: A Review

**DOI:** 10.3389/fbioe.2022.841389

**Published:** 2022-02-16

**Authors:** Hongyong Zhang, Guoguang Rong, Sumin Bian, Mohamad Sawan

**Affiliations:** CenBRAIN Lab, School of Engineering, Westlake University, Hangzhou, China

**Keywords:** network of neurons, lab-on-chip, microfluidics, microelectrode arrays, *ex vivo* studies, neurological disorders

## Abstract

Increasing population is suffering from neurological disorders nowadays, with no effective therapy available to treat them. Explicit knowledge of network of neurons (NoN) in the human brain is key to understanding the pathology of neurological diseases. Research in NoN developed slower than expected due to the complexity of the human brain and the ethical considerations for *in vivo* studies. However, advances in nanomaterials and micro-/nano-microfabrication have opened up the chances for a deeper understanding of NoN *ex vivo*, one step closer to *in vivo* studies. This review therefore summarizes the latest advances in lab-on-chip microsystems for *ex vivo* NoN studies by focusing on the advanced materials, techniques, and models for *ex vivo* NoN studies. The essential methods for constructing lab-on-chip models are microfluidics and microelectrode arrays. Through combination with functional biomaterials and biocompatible materials, the microfluidics and microelectrode arrays enable the development of various models for *ex vivo* NoN studies. This review also includes the state-of-the-art brain slide and organoid-on-chip models. The end of this review discusses the previous issues and future perspectives for NoN studies.

## 1 Introduction

The population that suffers from neurological disorders has been increasing rapidly in recent years. However, no therapy is available yet to effectively treat the majority of these neural diseases, including epilepsy, Alzheimer’s, and stroke (Pitkanen et al., 2015; Golyala and Kwan, 2017; Tang et al., 2017). Also, an increasing number of people have been diagnosed with depression and other mental illnesses, with no suitable medicine to choose from (Fiest et al., 2017). Such a situation makes it more urgent to understand the connections/networks between the brain’s neurons before carefully investigating the pathology of neural diseases. However, traditional methods such as electroencephalogram signals and functional near infrared spectroscopy do not provide sufficient accuracy and precision to study the brain. For example, electroencephalograms, the brainwave signals produced by active brain neurons, are recorded by electrodes around the head, forming neuroimages (Ramos-Argüelles et al., 2009; Kearney et al., 2018). A typical electroencephalogram recording device contains 20–40 electrodes. This indicates that one electrode records the entire signals from one particular large area but fails to focus on individual neurons with high resolution (Okamoto et al., 2004). To enable detailed *ex vivo* studies on networks of neurons (NoN), researchers are dedicated to building advanced lab-on-chip microsystems to better understand neurological diseases (Wong, 2011).

Microfluidic chips have been widely used to study neural cells and NoN and to build various *ex vivo* organ-on-chip models (Geraili et al., 2018; Holloway et al., 2021), including kidney (Asif et al., 2020), bone (George et al., 2018; Sheyn et al., 2019; Truesdell et al., 2020), heart (Kofron and Mende, 2017), liver (Maschmeyer et al., 2015), muscle (Morimoto et al., 2013), and brain (Kaneko et al., 2020). The advantages of microfluidics in high-throughput, high-efficiency integration, miniaturization, flexible architecture, and low costs in fabrication speed up the microfluidics-based *ex vivo* NoN studies (Sharma et al., 2013; Kou et al., 2016). Organ-on-chip devices, on the one hand, can better study the organs, and on the other hand, can be applied for drug discovery and toxicity tests to screen promising therapeutic drugs (Cao and Köhler, 2015; Osaki et al., 2018). Importantly, organs on chips can be customized as needed by manipulating cells inside the microfluidic chips (Yao et al., 2019).

Despite the advances in building advanced brain models with microfluidics, there remains a challenge in continuously recording the neural activities for real-time NoN studies *ex vivo*. Currently, patch clamp electrophysiology remains the gold standard for electrophysiological characterization of individual neurons from cells owing to its low noise and high resolution (Bébarová, 2012; Barthmes et al., 2014). This method however requires large equipment and complicated operations, which might damage the cells, and most undesirably, records the activity of a single cell, rather than the activities of cell populations each time (Accardi et al., 2016; Gao et al., 2021a). To address this challenge, microelectrode arrays (MEAs) have been proposed in recent years to monitor neural cell activities in real time (Newberry et al., 2016). Integration of cell culture with signals recorded in parallel by a well-designed MEA seems a more promising technique for *ex vivo* study of the NoN (Regehr et al., 1989; Johnstone et al., 2010). Compared to the patch clamp, MEAs can monitor thousands of cells on one single chip but cause no damage to cells. Also, MEA chips are small in size and easy to operate. One limitation of MEAs is that compared to a patch clamp that records intercellular potential, MEA records extracellular potential, which may lose some sensitivity. However, this limitation has been minimized recently through elaborate treatment on microelectrode surfaces (Choi et al., 2021). For example, the latest works showed that three-dimensional (3D) structured electrodes (Desbiolles et al., 2019; Saito, 2019; Yoo et al., 2020; Xu et al., 2021) enable MEAs to record the intercellular potential by membrane permeabilization or inserting electrodes into cells.

**FIGURE 1:**
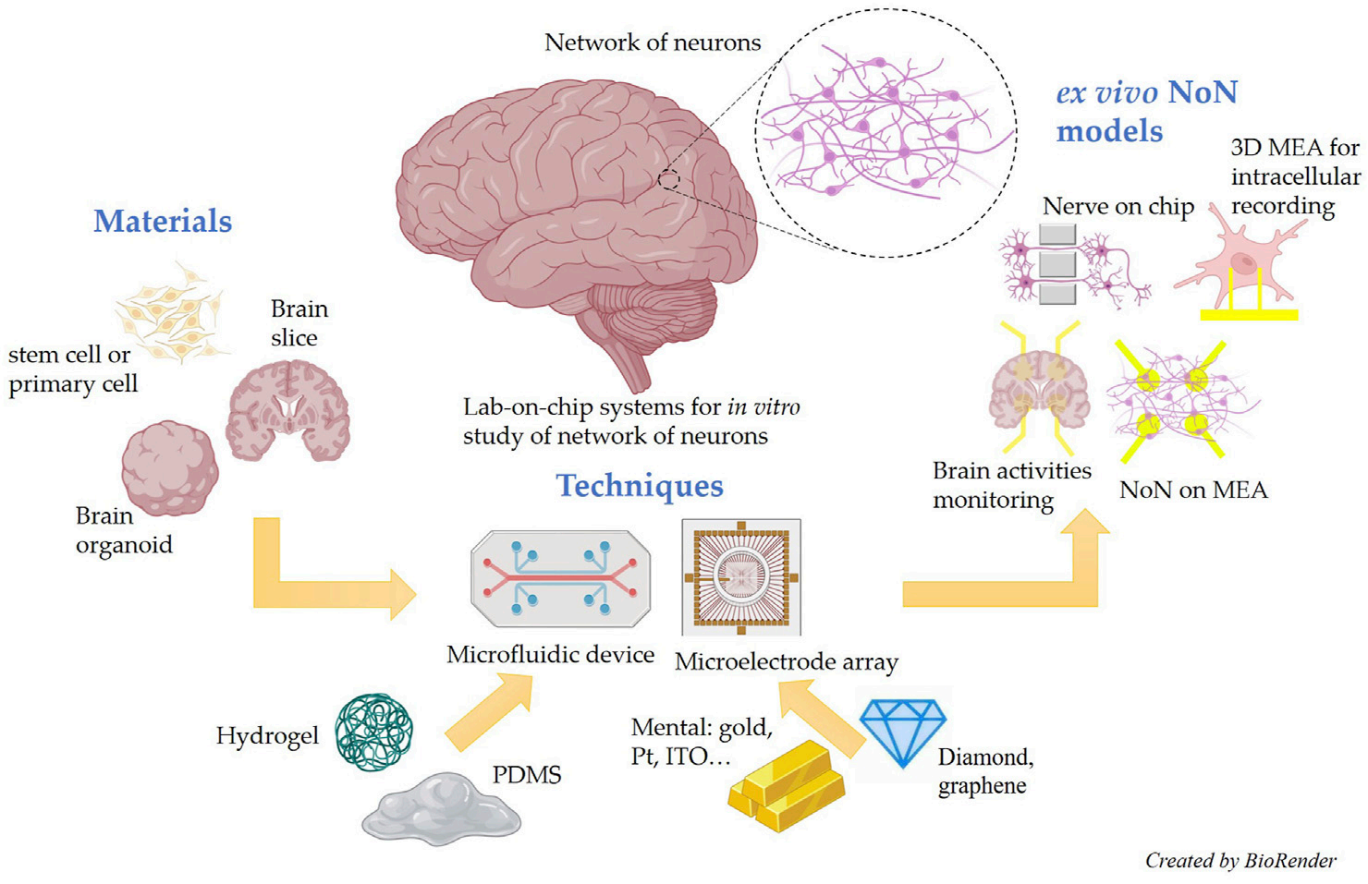
Schematic illustration of the state-of-the-art materials, techniques, and models for lab-on-chip microsystem-based *ex vivo* NoN studies.

This review aims to systematically summarize the latest advances in lab-on-chip microsystems for *ex vivo* NoN studies. For a clear understanding, we divide the review into three sections: advanced materials for *ex vivo* NoN studies, advanced techniques for *ex vivo* NoN studies, and advanced models for *ex vivo* NoN studies. The schematic illustration of this review is briefly shown in Figure 1.

## 2 Advanced Materials for *Ex Vivo* NoN Studies

### 2.1 Functional Biomaterials for Building Models

Human bodies cannot be studied directly for multiple reasons, including ethical and safety issues. To better study human diseases, human stem cells are a better alternative than animal-derived cells to build functional organ-on-chip models. This is more often the case in the fields of drug discovery and toxicity tests as there is a rather big gap between what happens in human bodies versus in animals. Previous research in sepsis showed that molecular mechanisms differ significantly between mice and humans, despite their similar disease phenotype (Seok et al., 2013). For brain-on-chip models, microfluidic chips with specific shapes have been proposed to culture various types of neural cells. The two main types of cells used in neural models are primary cells and stem cells (Grainger et al., 2018). Compared to stem cells, primary cells represent the organs inside the body better but are harder to obtain and proliferate slower. Also, primary neural cells are usually generated by animals, instead of humans. On the contrary, stem cells can reproduce themselves indefinitely and differentiate into any cell type in a suitable environment (Takahashi and Yamanaka, 2006; Takahashi et al., 2007). Stem cells, especially induced pluripotent stem cells (iPSC), are derived from human somatic cells and exhibit humanoid morphology. Disease models can be improved by using stem cells from patients without ethical considerations.

**FIGURE 2:**
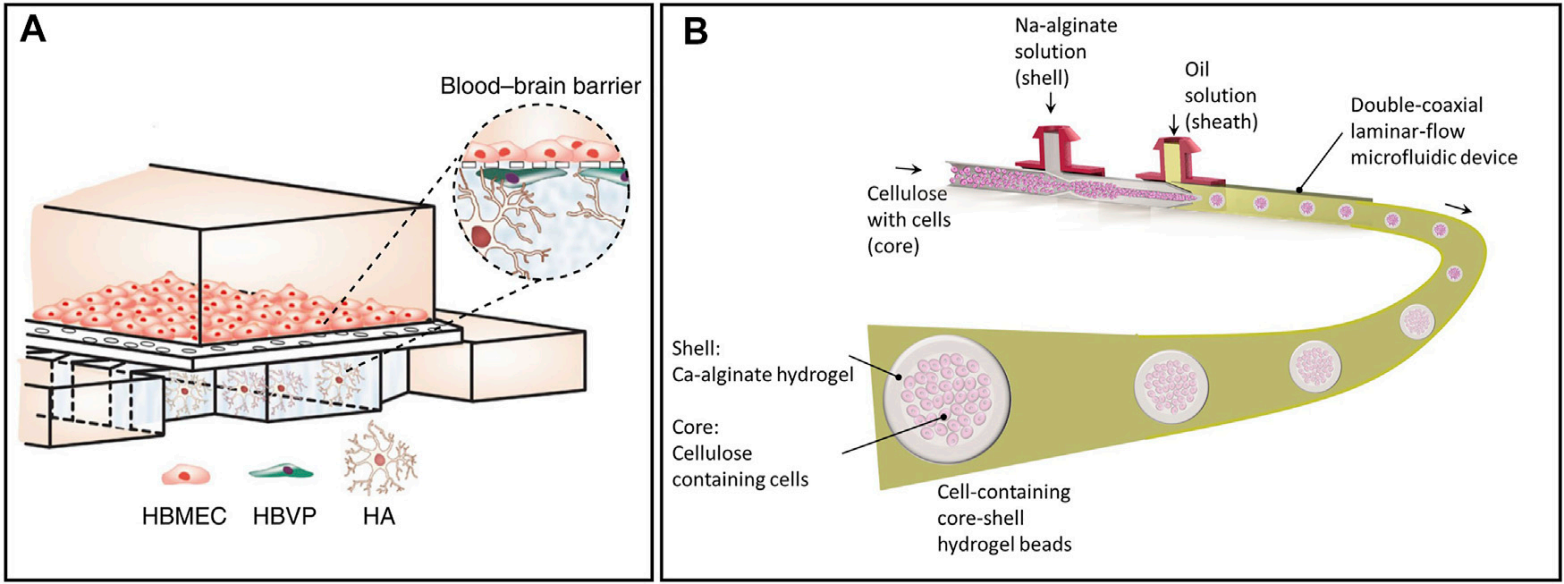
Representative advanced materials to build brain models *ex vivo.* **(A)** A blood–brain barrier model by endothelial cells, pericytes, and astrocytes (HBMEC: human brain microvascular endothelial cells, HBVP: human brain vascular pericytes; HA: human astrocytes) (Ahn et al., 2020). **(B)** A coaxial flow-focusing capillary-assembled microfluidic device to fabricate spherical hydrogel beads with cells in it (Liu et al., 2020).

Glial cells also play an essential role in neural activity since they support neural growth and biochemical transportation. Previous research indicated that glial cells could be related to many neurodegenerative diseases (Miller et al., 2004). For instance, astrocytes, the most abundant glial cells in the brain, regulate blood flow in the blood–brain barrier to keep the central neural system stable. Meanwhile, they are also quite sensitive to pathological triggers like infection and stroke. Regarding brain-on-chip models, co-culture of glial cells with neural cells can provide neurons with a better microenvironment and promote axon growth. Ahn et al. proposed a blood–brain barrier model by endothelial cells, pericytes, and astrocytes, as shown in Figure 2A (Ahn et al., 2020). The chip had two microchannels, combining a 2D endothelial monolayer with a 3D brain microenvironment, which mimicked the *in vivo* environment very well. This model was a promising tool for testing neural drugs and studying the blood–brain barrier in both physiological and pathological conditions.

Culturing neural cells *in vitro* helps us observe the developmental process of neural cells and the connections between neurons. Given that the brain is much more complicated than artificial NoN on a chip, it is impossible to be fully reconstructed *in vitro*. In addition, various neural cells, such as neurons, astrocytes, and microglia, are present in the human brain and interact with each other to play important roles in brain activity (Goshi et al., 2020). Artificial neural circuits help us understand the fundamental mechanisms of brain function but fail to achieve high-order functions such as emotion, memory, and recognition. In this respect, brain slides from animals can be an excellent alternative to establishing neural disease models as their structures are much closer to the human brain than planar NoN.

### 2.2 Functional Biocompatible Materials for Device Fabrication

The organ-on-chip model has become a hot topic in recent years as it mimics what happens inside the body to a great extent, with a low-cost and easy-to-fabricate process (Benam et al., 2015; Kim et al., 2015; Xu et al., 2016; Zhang et al., 2009). In contrast to traditional cells cultured in a petri dish, real organs are usually cultured in 3D structures. As such, scaffolds are required to culture the cells in 3D structures to mimic organs in the body (Murphy et al., 2017; Zhang et al., 2019). Hydrogel is one of the most popular scaffold materials in the 3D cell culture due to its excellent biocompatibility and softness. Additionally, the mechanical properties of hydrogen are similar to those of human organs under certain conditions (Pek et al., 2010; Zhu and Marchant, 2011; Mahoney and Anseth, 2006). Other materials like fibrous and solid porous are also popular for cell culture use for various applications (Hayman et al., 2005; Cao et al., 2009). Liu et al. proposed a coaxial flow-focusing capillary-assembled microfluidic device to fabricate spherical hydrogel beads with cells in them, as shown in Figure 2B (Liu et al., 2020). Alginate, one kind of hydrogel that could be solidified with calcium iron, was used as the shell of the sphere. Cells inside the spheres could grow well due to the permeability, biocompatibility, and softness of hydrogel. It was a promising tool that could be used to culture brain organoids. Some other hydrogels, such as matrigel and collagen, are mainly used to coat the surface of culture dishes or chips to promote cell adherence and growth.

As learned from the abovementioned messages, MEA is widely used to record signals from cells in a given small area. To fabricate MEA, gold is considered an ideal material because of its high conductivity and biocompatibility. For fluorescence immunoassay experiments that require high transparency of the substrate for better observation, indium tin oxide glass, as a combination of electrical conduction and optical transparency, is more often used in fabricating such microelectrodes. However, the relatively high impedance to obtain high transparency leads to high background noise and a low signal-to-noise ratio. Poly(3,4-ethylenedioxythiophene) polystyrene sulfonate (PEDOT: PSS) (Koutsouras et al., 2017; Kshirsagar et al., 2019; Susloparova et al., 2021) is a new interfacial material for modern bioelectronics and is better than ITO in some aspects, such as conductivity and flexibility. It is still facing some challenges like longevity and multichannel (Liang et al., 2021). As such, new materials have been developed in recent years to improve the electrodes, such as boron-doped diamond (McDonald et al., 2017) and graphene (Koerbitzer et al., 2016; Thunemann et al., 2018). Their excellent properties in better transparency and higher conductivity make them attractive for various applications in the near future.

For microfluidic chips, the most common material is polydimethylsiloxane (PDMS) because of its excellent elasticity, optical transparency, and biocompatibility (Wang et al., 2014). It also has high resolution on microstructure fabrication and has been widely used to build microchannels and microwells by molding. The basements are usually silicon wafers with microstructures on them. If the precision demand is not high, 3D-printed soft lithography is a good choice due to its low cost and ease of fabrication (Wu et al., 2017).

## 3 Advanced Techniques for *Ex Vivo* NoN Studies

### 3.1 Microfluidic Systems

Micro-/nano-fabrication techniques such as photoetching and thin film deposition have been developing rapidly recently. Microfluidic systems are getting popular in the bioengineering field for neural degeneration disease modeling, neuroprotective mechanism modeling, and neural circuit establishment (Lassus et al., 2018). Single-cell manipulation can be achieved in the microfluidic chip, and microenvironment around cells can be controlled precisely, making microfluidic systems an up-and-coming tool to conduct neural study (Chen et al., 2011).

#### 3.1.1 Cell Manipulation for Single-Cell Study

The advance of modern technologies is overturning some conclusions drawn in previous studies. In our traditional cognition, only nerve cells are involved in neural communication. Increasing shreds of evidence show that gliocytes also play an essential role in neural communication, although the effect of gliocytes on the whole process remains to be understood (Liu and Wang, 2016; Zhang et al., 2016). In this case, cell manipulation techniques provide a promising way to study cell communication at a single cell level (Yun et al., 2013). Fluorescence-activated cell sorting is a classic method for single-cell analysis in biological labs, which requires expensive equipment and complicated sample pretreatment. The character of instantaneous detection made this technique impossible to achieve long-term monitoring. In this respect, the microfluidic chip is a promising platform for cell manipulation and long-term continuous monitoring due to its biocompatibility, design, and low cost. Various microfluidic chips have been designed to manipulate cells in the past 10 years (Sen et al., 2013; Chu et al., 2015; Miled et al., 2015; Zhao et al., 2020).

**FIGURE 3:**
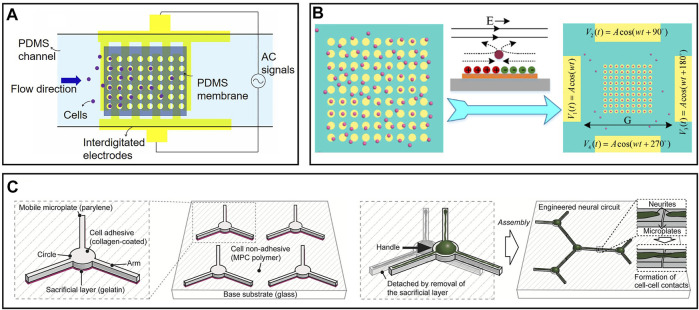
Representative cell manipulation on microfluidic chips: **(A)** Cell manipulation chip based on dielectrophoresis via interdigitated electrodes. Cells were attracted by non-uniform electrical field and fall into microwells (Farasat et al., 2021). **(B)** Cell manipulation based on induced charge electroosmosis. Particles moved with the flow and trapped in the center of electrodes (Wu et al., 2016). **(C)** Cells would move to cell-adhesive mobile plate automatically. Neural circuits could be establishment by manipulating these microplates (Yoshida et al., 2016).

One of the most popular methods for manipulating cells is dielectrophoresis (DEP). Neutral particles will polarize under a non-uniform electric field and be subject to dielectrophoretic force. After that, they move to a position where electric field is stronger or weaker, depending on the permittivities of the cells and solution (Green et al., 2000). The equation of DEP can be expressed as follows (Giugiulan et al., 2014):

FDEP=2π(dc2)3εfRe[CM(ω)]∇|Erms|2

$$CM\left(\omega \right)=\frac{\varepsilon_{c}^{*}-\varepsilon_{f}^{*}}{\varepsilon_{c}^{*}+2\varepsilon_{f}^{*}}$$

where d_c_ is the diameter of cells or neutral particles; E_rms_ is the root mean square of electric field intensity; and $\varepsilon_{c}^{*}$ and $\varepsilon_{f}^{*}$ are the complex permittivities of cell and fluid. Compared to traditional cell manipulation methods including gravity and magnetic fields, DEP’s advantages are conspicuous: high efficiency, label-free detection, low cost, design, and high throughput. Farasat et al. proposed a cell-trapping microfluidic chip based on dielectrophoresis *via* interdigitated electrodes, as shown in Figure 3A (Farasat et al., 2021). An AC signal was applied to the interdigitated electrodes to form a non-uniform electrical field on the PDMS membrane. Cells would be attracted and fall into the microwells. According to this principle, cell pairing could also be achieved. Wu et al. proposed a microfluidic chip for high-throughput single-cell pairing based on positive DEP (Wu et al., 2017). Two groups of planar interdigitated electrodes were fabricated in tandem to produce a non-uniform electric field in tandem. Hela cells with green and red fluorescence were used as cell A and cell B for pairing. Group A interdigitated electrode trapped cell A into a 16.5 µm well, the average diameter of a Hela cell, to make sure that only one single cell was trapped into one well. Cell B was trapped in the other well later. After being trapped in a big well, Cell A and Cell B were isolated and eventually connected together through a small pushing process. This chip was shown to be a desirable platform to investigate cell communication in large quantities, attributed to its high throughput (more than 2,400 pairs in a 1 × 1.5 cm area) and high pairing efficiency (up to 74.2%).

Recently, induced-charge electroosmosis has also gained increasing attention in manipulating cells (Nam et al., 2015; Ren et al., 2015; Ren et al., 2016). Electroosmosis and electrophoresis achieve similar effects, but the mechanisms behind them are different. For electrophoresis, particles are driven by electrophoresis directly, and the solution is stable; for electroosmosis, particles remain stationary relative to the solution as driven by electroosmosis. Wu et al. (2016) proposed a simple cell capture device on MEAs based on induced charge electroosmosis, as shown in Figure 3B. Four driving electrodes are energized with four sine signals with different phases to create a rotating electric field. On the surface of each microelectrode in a rotating electric field, flow vortices are formed that transport particles to the center of the electrode. Compared to the DEP method mentioned above, this device is easier to fabricate and infinitely expandable. Cells can be trapped in any place as desired in the rotating electric field by simply adding a microelectrode. The limitation of this device lies in its low efficiency as particles flow with the solution.

Except for passive movement driven by external forces, cells can sometimes migrate to cell-adhesive areas from non-cell-adhesive areas spontaneously. Various shapes of cell-adhesive patterns have been designed on microfluidic chips to control the growth of neurons or axons (Merz and Fromherz, 2002; Onoe and Takeuchi, 2008; Fricke et al., 2011; Roth et al., 2012; Benzina et al., 2014). In addition, pieces of evidence show that circular-shaped and linear-shaped micropatterns have different extents of adhesion to neural soma and neurites. Yoshida et al. proposed an exciting method to establish freely combinable neural circuits *in vitro* via a modified mobile microplate, as shown in Figure 3C (Yoshida et al., 2016). These microplates have a circular body and several linear arms, corresponding to neural soma and axons, respectively. They were placed on a large planar cell-repulsive substrate so that cells could spontaneously migrate to the mobile microplates. The mobility of microplates enabled neural circuits to be built up by moving their arms close by. Compared to traditional fixed cell-adhesive pattens, this method is extensible, controllable, and more efficient as microplates without cells can be easily removed from the basement.

We should notice that most of the cell manipulations based on electrical fields require tuning of the conductivity of the solution. Increased conductivity may turn DEP from being positive to being negative. Conductivity also determines which electrophoresis and electroosmosis are dominant. Commonly, a small number of irons are added to the solution to adjust the conductivity, and sucrose is used to adjust the osmotic pressure. Also, any changes in the solution or external electric field may affect cells, especially neural cells. In order to minimize the external influences on cells, microstructure-based cell manipulation, which does not require any electrical field or adjustment of conductivity, has become great interest to researchers (Carlo et al., 2006; Frimat et al., 2011; Pang et al., 2020). Such microstructures include cup-shaped traps (Di Carlo et al., 2006), micropillars (Tan et al., 2009), and pneumatic valves (Kim and Kim, 2014). A surface acoustic wave, as a mechanical wave, is also used to arrange cells without the requirement of solution conductivity (Collins et al., 2015). Acoustic wells with minimum local potential are generated in the acoustic field where particles fall into place. The density of acoustic wells can be adjusted by changing the ratio of acoustic wavelength to particle diameter. This method not only avoids direct contact between cells and microstructures but also has no requirements on the properties of particles, showing high potential in cell manipulation.

#### 3.1.2 Microenvironment Control on a Chip

**FIGURE 4:**
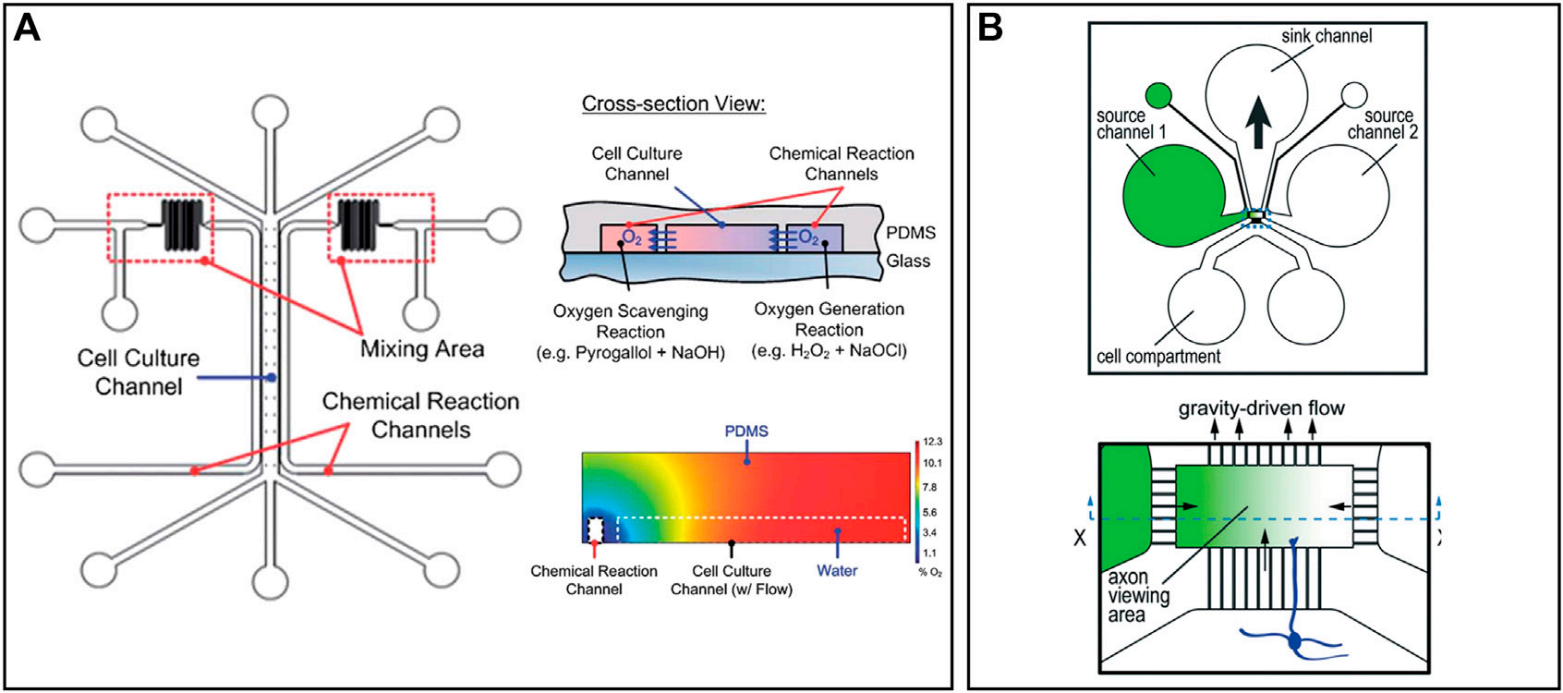
Representative microenvironment control and MEAs on chips: **(A)** A microfluidic device that can precisely generate an oxygen gradient in the microchannel by chemical reactions (Chen et al., 2011). **(B)** Five microfluidic chambers connected by microtunnels to create a solution concentration gradient. Axons grow through microchannels and effected by the concentration gradient (Taylor et al., 2015).

The environment close to the cells is critical as one tiny factor may cause remarkable effects on cells, particularly for stem cells. The microenvironment decides the direction of cell differentiation, cellular vitality, and lifetime. Plenty of methods have been proposed for long-term cell culture and maintaining a stable extracellular microenvironment (Choi et al., 2015; Oyama et al., 2018; Mun et al., 2020). Scientists may change the extracellular environment deliberately to build up a certain disease model in order to show how the changes in the microenvironment in the brain cause the onset of a disease (Osaki et al., 2018). Stroke, the most significant cause of adult disability, is caused by a lack of blood or oxygen in the brain. Still, no effective neuroprotective therapy has been proposed to treat stroke (O'Collins et al., 2006). Chen et al. proposed a microfluidic device that can preciously generate an oxygen gradient in the microchannel by chemical reactions, as shown in Figure 4A (Chen et al., 2011). A PDMS with high gas permeability was patterned by a soft lithography technique and adhered to a glass substrate. Three microchannels were formed and separated by thin PDMS membranes with a thickness less than 50 µm. Two channels on the side were used to produce and scavenge oxygen, respectively, by chemical reactions. One wider channel in the middle was used for cell culture, where an oxygen gradient is generated and stably maintained. Compared to conventional gas control devices that require complicated equipment and a large volume of gas, microfluidic devices are easy to build, small in size, low cost, and energy efficient. Besides, it avoids direct contact between cells and chemicals, which minimizes the effects of the latter. Additionally, this device can produce a gradient of other gases by changing the reaction chemicals, enhancing its rationality and usability.

Solution concentration gradient generation is a little different from gas because liquid cannot go through the PDMS. Taylor et al. designed a microfluidic gradient chamber to study the influence of growth factors on mouse neocortical neurons, as shown in Figure 4B (Taylor et al., 2015). A total of four channels were built around the axon viewing area, where a gradient of growth factor concentration was produced. Left and right channels were used to input culture solutions with different concentrations of growth factors. The height of the axon viewing area is much smaller than other channels, which decreases the flow rate to stabilize the gradient. The solution will flow out through the upper channel due to gravity. Neural cells were cultured in the channel below and axons grew through the microgrooves, which reduced the influence of the initial growth direction of axons. Compared to traditional methods with a concentration gradient in different petri dishes, this microfluidic chip simplified the operation and better controlled the initial conditions of axons.

### 3.2 Microelectrode Array Systems

Commercially available planar MEAs are 8 × 8 square which meets the requirements of the majority of experiments for neural research (Furukawa et al., 2013; Rastegar et al., 2019; Gao et al., 2021b). However, 64 electrodes are not sufficient when studying a large-scale NoN study that involves complex connections between cells. Large-scale MEA recording systems have been developed as such to better study NoN over the past few years (Lott and Hoy, 2008; Laurent et al., 2009; Maccione et al., 2012). Due to the limitations on the size of microelectrodes and chips, it is infeasible to match each electrode with its own amplifier chain and digitizer. In this case, time-division multiplexing is widely used for high-throughput recordings (Berdondini et al., 2009). However, traditional time-division multiplexing has a low signal-to-noise ratio when applied to thousands of electrodes. David et al. proposed a CMOS electronics-based super large-scale MEA chip that contains 65,536 electrodes (Tsai et al., 2017). Compared to traditional methods, this large-scale MEA has high density, low noise, and, particularly, does not require antialiasing filters for each channel to overcome scaling limitations. Furthermore, thin-film-transistor (TFT) technology has been widely used in display fabrication and has also been used to fabricate MEAs in recent years (Shaik et al., 2017). Compared to the traditional homemade MEAs, although the circuit design could be more complex, it is easier to get and has stable quality. Some elaborate TFT electrode array chips can achieve cell manipulation *via* dielectrophoresis (Tixier‐Mita et al., 2019).

Noise could be an issue during the electrophysiology recordings since the electrical signals produced by cells are rather small. Except for the system noise, the majority of the noise comes from the change of the ion environment near electrodes (Hai et al., 2010; Mateus et al., 2019). Thus, increasing the contact area between cells and electrodes is a promising strategy to increase the signal-to-noise ratio. Abbott et al. proposed a nanoelectrode array coated with platinum-black, which not only increased the surface area but also lowered the impedance of electrodes (Abbott et al., 2020a; Abbott et al., 2020b). A cell–electrode interface model was also built, as shown in Figure 4C. Additionally, intracellular signals can be detected through membrane permeabilization, induced by a small faradaic current. In this way, tiny signals can be recorded, which is the key to investigating the synaptic connectivity between neurons. One individual electrode performs similarly to the patch clamp, but the entire electrode array is scalable, subminiaturized, and of high density, which is beyond the reach of the patch clamp. Cell stimulation can also be achieved in each electrode by a small current. The stable and precise recording makes network-wide mapping of neurons possible, which certainly promotes further research into the human brain *ex vivo*.

Not only electrical signals can be used for neural recording and stimulation, but optical signals and chemical signals can also represent the activities of neurons. Through optogenetic modification, inserting light-sensitive genes into cells, cells can be stimulated by different colors of light (Yoo et al., 2020). Meanwhile, neural activity can be reflected by fluorescence intensity in some aspects. In addition, the optogenetic technique has already been used *in vivo* several years ago to control the brains of mice, creating the most famous mouse that has been featured in countless top journals (Nabavi et al., 2014). The fundamental principle of chemical recording is that neurons release neurotransmitters when they communicate with each other. Various biosensors have been proposed to monitor these chemicals in real time (Su et al., 2020). Also, some chemicals can be used to stimulate neurons. Amanda et al. proposed a bioelectronic neural pixel to stimulate neurons by chemicals and record their activities simultaneously with PEDOT:PSS electrodes because it has both conductivity and permeability (Amanda et al., 2016). A cation exchange membrane was used to deliver chemicals, and gold was used to conduct electrical signals. This bioelectronic neural pixel is a potential platform to study the effects of different neurotransmitters on neurons.

Furthermore, culturing cells on MEAs for the long-term is essential for NoN development, as axons and connections between cells take time to grow. The major obstacle to long-term culture is infection from the environment, which can be minimized with the use of a clean environment and standard operation. Previous research demonstrated that hyperosmolality, caused by medium evaporation, is the second reason for neuron apoptosis (Potter and DeMarse, 2001). Many devices for long-term culture of neural cells have therefore been equipped with their own gas supply system or membrane-sealed chamber to avoid evaporation.

## 4 Advanced Models for *Ex Vivo* NoN Studies

### 4.1 Microfluidic-Based Models for *Ex Vivo* NoN Studies

**FIGURE 5:**
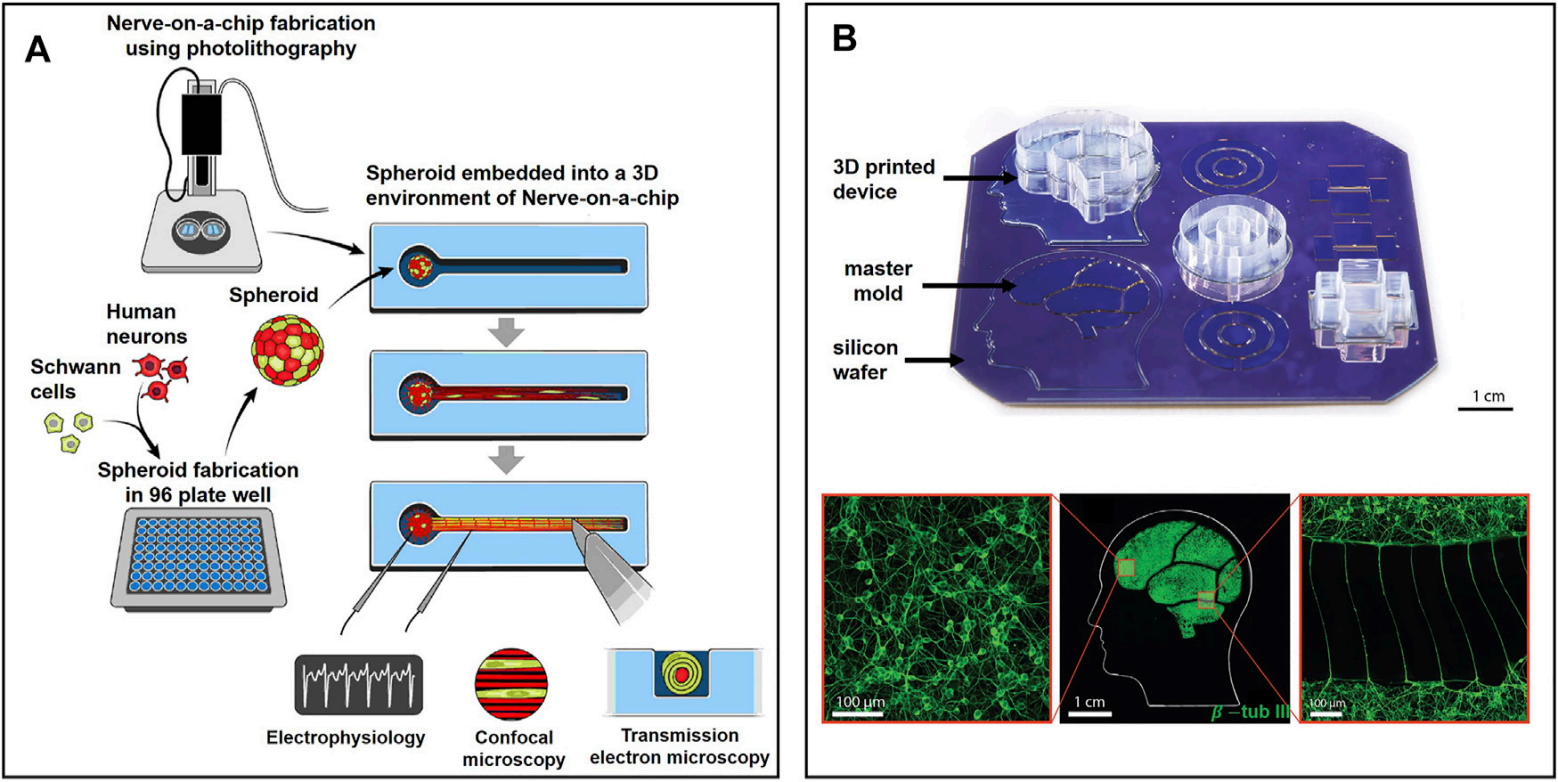
Representative state-of-the-art microfluidic-based models for *ex vivo* NoN studies: **(A)** Lollipop-shaped container made by dual hydrogel to develop myelinated peripheral nerve model by human iPSC (Sharma et al., 2019). **(B)** Micro-chambers and -tunnels made by 3D-printed soft lithography to study Parkinson’s disease. Neurons were connected by axons through microtunnels (Kajtez et al., 2020).

Kramer et al. fabricated a lollipop-shaped container using dual hydrogels to develop a myelinated peripheral nerve model, as shown in Figure 5A (Khoshakhlagh et al., 2018; Kramer et al., 2020). Neural cells and gliocytes are co-cultured in a spheroid microplate to form a cell sphere that will be moved to the circle part of the container. Axons will grow through the channel in a few weeks. In order to mimic the human brain to a great extent, researchers also applied human iPSC to build a peripheral nerve model (Sharma et al., 2019). It is a desirable platform to study neurodegenerative diseases and perform drug-related tests. After four weeks of culture, a patch clamp was used to monitor the electrophysiology of their nerve model, including nerve conduction velocity and amplitude, which are gold standards for evaluating chemotherapy-induced peripheral neuropathy (Brewer et al., 2016; Stagg et al., 2016). Meanwhile, the nerve model was challenged by six types of drugs with or without neuropathic properties, and the results were as expected, demonstrating its reliability in drug tests.

It is nearly impossible to decrypt the brain even with modern state-of-the-art technologies due to the complexity of our brain. In this case, guiding neurites by microtunnels between two isolated microfluidic regions provides an alternative way to study the NoNs (Jean-Michel et al., 2011; Renault et al., 2015; Samson et al., 2016a; Samson et al., 2016b; Renault et al., 2016). Such type of microfluidic device can be used to study synaptic plasticity, disease processes, and communication between different brain regions that are related to certain neural diseases. For example, in a generalized seizure, epileptic seizures spread to the whole brain, while in a focal seizure, the seizures stay within a certain small area (Fisher et al., 2017). The effects of the central nervous system on other tissues, such as muscles and blood vessels, can also be studied on the chip by guiding axons to germinate in the corresponding tissues through microchannels while preventing cell migration (Gilbert-Honick et al., 2020). However, the development of this technology is limited due to the complicated operations of soft lithography, which generally includes the pouring of liquid PDMS into a micro-structured wafer and tearing it off manually once solidified. Such a process can damage the microstructures and limit the design options, as structures in 3D and with large aspect ratios cannot be molded.

Three-dimensional-printed soft lithography provides a better option to fabricate complex microfluidic structures, as PDMS can be extruded everywhere on the chip with high resolution. Kajtez et al. proposed a prototype composed of several chambers which are connected by microtunnels (Kajtez et al., 2020). Gasket ink and compartment ink were developed for extrusion in a master mold to generate tall “walls” between chambers, as shown in Figure 5B. Human neural stem cells were cultured on the microfluidic chip to study Parkinson’s disease *via* modeling of nigrostriatal pathways by guiding the growth of dopaminergic projections through microchannels. Compared with traditional lithography methods, 3D-printed soft lithography has high precision and high resolution and greatly simplifies the fabrication steps. These advantages make it widely applied for printing the framework for cell growth and cell communication (Achille et al., 2021). Three-dimensional bioprinting was developed on the basis of 3D printing. Cells and scaffolds are generally made of polymers and extruded from a nozzle and cultured in 3D (Elalouf, 2021; Jamee et al., 2021). Many organs, such as bone (Arrigoni et al., 2017; Kim et al., 2021; Alcala-Orozco et al., 2022), lung (Hu et al., 2021), and skin (Gao et al., 2021c), have already been generated by 3D bioprinting, showing plenty of potential in the future. Cell delivery (Hwang et al., 2022) and tumor modeling (Samadian et al., 2021) can also be achieved by bioprinting. Today, 3D printing is still in its initial stage and requires much effort to improve, especially in cell co-culture technique, bioink properties, and fabrication methods. Nevertheless, the great benefits of flexible design and rapid prototyping will certainly ensure a bright future for 3D bioprinting in the coming years.

Except for building neuron models in microfluidic systems, the system can even perform like a neuron after elaborate design. Levi et al. proposed a microfluidic neuron that is similar to a biological neuron (Levi and Fujii, 2016). Each microfluidic neuron had a chamber representing an intracellular environment. Chambers were connected by microtunnels, representing axons that transmit signals. The exchange of irons was controlled by Quake valves and selective ion permeable membranes in microtunnels. Thus, the membrane potentials were formed and measured by integrated electrodes. It could be a promising tool to explore in neuromorphic engineering.

### 4.2 3D MEA-Based Models for *Ex Vivo* NoN Studies

**FIGURE 6:**
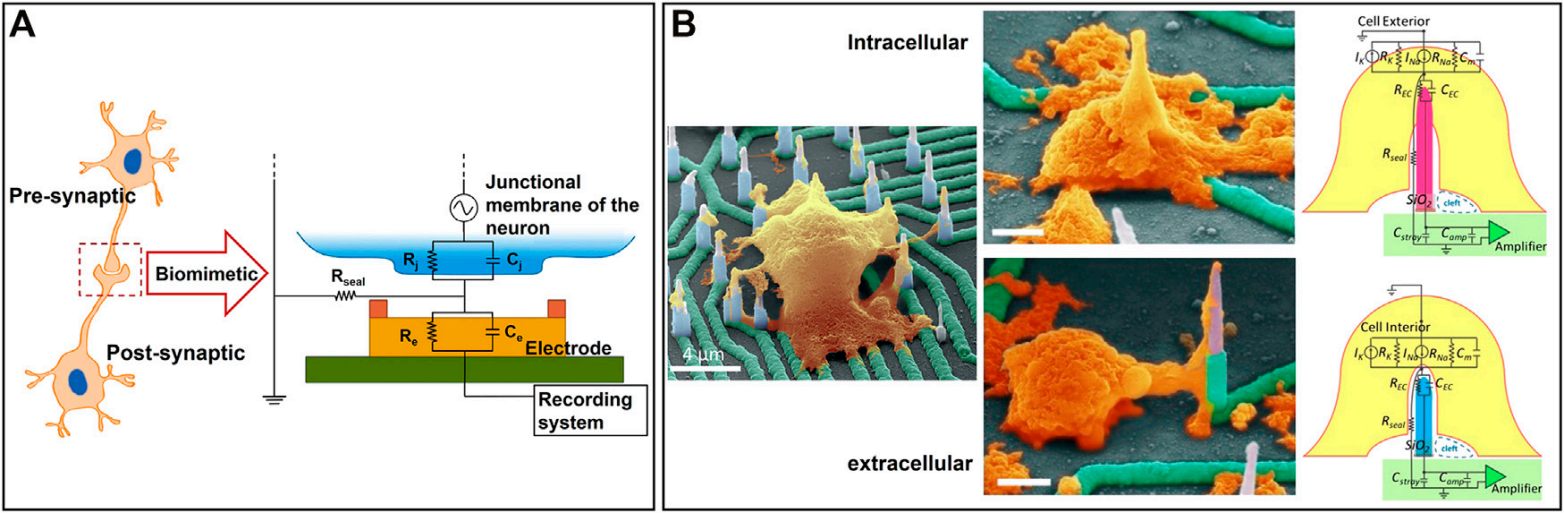
Representative state-of-the-art 3D MEA-based models for *ex vivo* NoN studies: **(A)** A bio-mimicking and high-resolution planar MEAs for long-term neural recordings (Wijdenes et al., 2016). **(B)** A 3D high-density nanowire array for both exocellular and intracellular signals recording (Liu et al., 2017a).

Large-scale MEAs can monitor the neural activities of the whole NoN, but it remains challenging to attain high resolution and to record potential changes in a single cell. Decreasing the size of the electrode is an alternative solution to achieve high-resolution recording. In this way, noise can also be reduced as the entire surface of the electrode is covered by cells, which avoids any potential negative effects from iron in solution. Inspired by the morphology of a synapse, Wijdenes et al. proposed a novel bio-mimicking and high-resolution planar MEAs for long-term neural recordings, as shown in Figure 6A (Wijdenes et al., 2016). Several electrodes were applied to record one single cell simultaneously, increasing the resolution by 14 times as compared to the traditional planar electrode. Additionally, the edge of each electrode was modified with gold nanostructures to increase the contact area between neurons and electrodes. This nano-edge structure prevented current leakage into the environment, which reduced the noise and obtained high-quality signals.

In order to further improve the signal quality and the sensitivity of MEAs, 3D microstructures can be modified on the top of the electrodes, such as nanostraws (VanDersarl et al., 2012), nanowires (Robinson et al., 2012), and nanopillars (Xie et al., 2012). The main advantage of 3D structured electrodes is that intracellular action potential can be detected, which reflects the information of ion channels and synapses of a single cell (Mechler et al., 2011). Although some of the aforementioned planar electrodes can record intracellular signals by electroporation (Abbott et al., 2020a; Buzsáki, 2004), they may disturb spontaneous cell activities at the same time as the membrane is damaged. Also, such electrodes require complicated circuit design and operation. In this case, 3D structured electrodes provide a harmless, simple way to measure high-quality exocellular and intracellular potential changes. Liu et al. generated a 3D high-density nanowire array to record both exocellular and intracellular signals (Liu et al., 2017a). Electrical circuit models were built, as shown in Figure 6B. Planar circuits were made of Ni and patterned by photolithography and electron beam lithography on top of a transparent sapphire substrate. A Ni mask and plasma etch technology were used to generate the Si nanowire with a proper height of 10 µm and SiO_2_ being deposited as the insulating layer. Human iPSC were cultured on the nanowire and induced into neurons. Nanowire electrodes under the soma entered into cells during the growth phase to record intracellular signals; electrodes near neurites were able to record exocellular signals. Casanova et al. fabricated nanowire probes on a chip through a CMOS-compatible large-scale fabrication process based on conventional lithography tools (Casanova et al., 2018). It could achieve a high surface-to-volume ratio and have a large signal-to-noise ratio. Compared to planar electrodes, 3D-empowered electrodes led to improved signal quality with reduced background noise and increased signal amplitude.

Gu et al. designed an elaborate scalable field-effect transistor (FET) array that can switch between 2D and 3D as shown in Figure 6C (Gu et al., 2021). The process of transformation is accomplished by compressive buckling technique, a pre-strained elastomer substrate was used to buckle the 2D electrode into 3D. It was able to record the signals both from planar NoN and 3D tissues. Due to the fact that the distance between two electrodes was smaller than the length of cardiomyocytes, it could also record the intercellular signal conduction. They found that the conduction velocity of intracellular signals in cardiomyocytes is about five times the conduction velocity of intercellular signals. It is a very promising platform to study the signal conduction pathways and screen drugs.

Despite the limited damage of 3D electrodes on cells, some effects may still happen to cell electrophysiology. Mateus et al. proposed a mushroom-liked 3D electrode array to further improve electrophysiology recordings (Mateus et al., 2019). The mushrooms were wrapped in cells so that, on the one hand, signal quality was much higher than that of planar electrodes; on the other hand, only very limited noise was obtained from the environment since all the surface of the electrode was covered by cells. Despite their capability of measuring exocellular signals only, 3D electrodes were totally non-invasive and performed better than most planar electrodes. Topography effects on neural cells were demonstrated in this study as well. Besides, this chip is compatible with commercial MEA data acquisition systems, which shows excellent application value.

### 4.3 Microfluidic MEA Combined Models for *Ex Vivo* NoN Studies

Synapse plays an important role in neural communication since it can transmit the excitatory impulses produced by the neuron body to other neurons or effectors (Lines et al., 2017). MEAs can record most electrical activities from somas, but signals from axons are hard to measure due to the limited diameters of axons. In this case, the microfluidic device is a powerful tool to enhance the recording of signals from neurites (Narula et al., 2017). Shimba et al. modified microtunnels on top of MEAs to limit the neurites elongation (Shimba et al., 2019). Laminin and poly-L-ornithine (PLO), which can promote the growth of neurons, were coated on the surface of tunnels to induce the neurites. Each tunnel contains a microelectrode inside to detect the neural activity form neurites, as illustrated in Figure 6D. Compared to neurons on conventional MEAs, neurons on this advanced chip achieve longer neurites elongation. Recording of neural activities was significantly enhanced, as a result of significantly increased number of neurites per electrode. Importantly, neurites in the microtunnels can sustain for more than 1 year, demonstrating high potential of neural long-term culture and monitoring.

Neurodegenerative disease models can be potentially established on the MEA-microfluidic combined chip, as it can record signals from neurons and manipulate cells simultaneously. Interestingly, as only neurites can be induced to go through the microtunnels, the combined chip is a powerful tool to study communication between neurons and body cells. For example, scientists have classified several kinds of epilepsy, and one of them is called focal epilepsy, indicating that only part of the brain, rather than the whole area of it, suffers from seizures (Shorvon, 2011; Fisher et al., 2017). In order to study the connection between brain regions with and without seizure, Anssi et al. designed a platform that contains three compartments with microtunnels to connect them (Pelkonen et al., 2020). The microtunnels prevented liquid exchange, and therefore liquids were isolated within their own compartment. Human iPSC were cultured in all three compartments and induced to neurons to represent different brain regions. Neurons in three compartments were connected by axons through microtunnels. The convulsant and kainic acid were added to one compartment to mimic focal epilepsy, while the other two compartments were not treated with any drug. In general, the combination of MEAs with a microfluidic device empowers MEAs with excellent performance and amazing functions.

### 4.4 Brain Slides-On-Chip Models for *Ex Vivo* NoN Studies

Evidence has shown that hippocampal slices induced the onset of epilepsy spontaneously after several days of culture *ex vivo* (Dyhrfjeld-Johnsen et al., 2010). Jing et al. cultured the organotypic hippocampal slices in a petri dish to study the effects of the culture solution on spontaneous epileptogenesis (Liu et al., 2017b). A tungsten microelectrode was placed in a hippocampal slice to record extracellular field potential, and different culture solutions were added to the dish. Results showed that improvements in culture medium could prevent apoptosis of the brain to some extent, but seizures always happened. However, one electrode cannot detect the spikes in different parts of the hippocampal slice. Two years later, the author designed a microfluidic MEA-combined chip with simplified linear electrodes made by PDMS to better record the seizure of hippocampal slices (Liu et al., 2019). Compared to the traditional tissue slides on MEAs (Scott et al., 2013), this integrated chip has six culture wells on one plate to achieve high-throughput drug discovery and better control the environmental variants.

**FIGURE 7:**
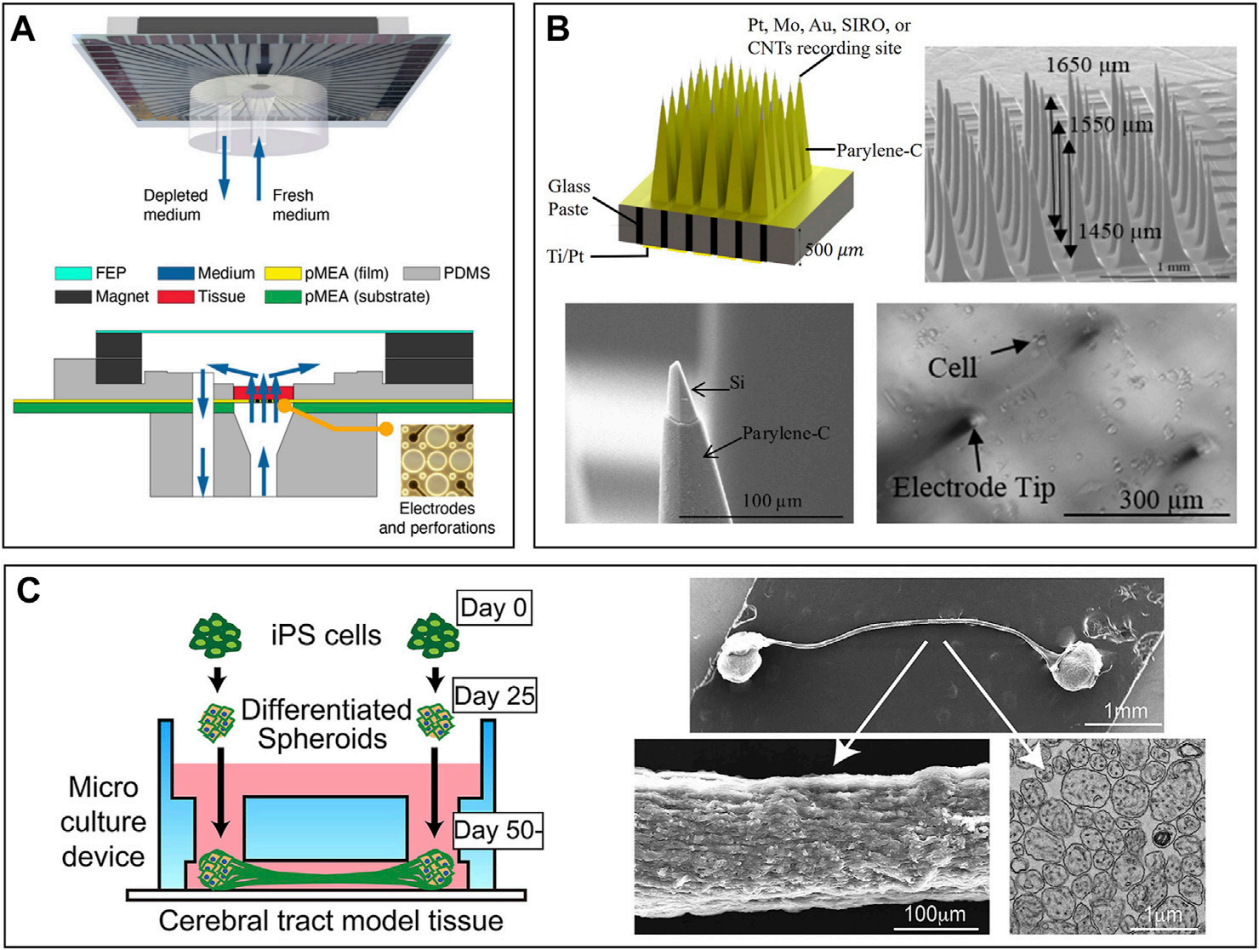
Brain slides and organoid-on-chips based models for *ex vivo* NoN studies: **(A)** Long-term perfusion, imaging microfluidic device based on MEA (Killian et al., 2016). **(B)** Micro-probe arrays used *in vivo* to record cell activity in the brain (Ghane Motlagh et al., 2016). **(C)** A microfluidic chip to build a cerebral tract model connecting two cortical regions. Organoids were first cultured in spheroid chambers and axons would grow through the microchannels (Kirihara et al., 2019).

During the recording process on MEAs, thick brain slides (>500 µm) may face issues of lack of oxygen, nutrition, and waste removal. These issues prevent us from studying the chronic toxicity of drugs and the subsequent progression of epilepsy. To address them, Killian et al. designed a long-term perfusion and imaging microfluidic device on the basis of MEAs, as shown in Figure 7A (Killian et al., 2016). Perforated MEAs were used to record signals from brain slices and to perfuse the culture medium from the bottom. Meanwhile, culture solution was extracted, with a volume equal to perfusion, to keep the environment stable. Compared to culture in dish methods, the tissue slides survive much longer and facilitate long-term experiment conductance.

### 4.5 Organoid-On-Chip Models for *Ex Vivo* NoN Studies

An organoid, a 3D cultured cell sphere, has characteristics between a mature brain and a planar NoN. Research demonstrated that neurons in 3D culture could have longer axons than in 2D culture, indicating that the function of neurons is limited in planar NoNs (Kong et al., 2021). In contrast to the real brain, which has blood vessels to provide nutrition for neurons, the organoid is made of neural cells only. Thus, one big issue in organoid culturing is the lack of oxygen in the center. Although some research shows that co-culture organoid with vasculature can promote the maturation and differentiation of neurons, the oxygen issue still remains (Isshiki et al., 2020). Thus, the organoid is much more complex than planar NoN due to its 3D structure but simpler than the human brain due to its limited functions. MEA was used to record the neural activities on the surface of organoid. The perfusion microfluidic devices previously introduced can also be used for culturing organoids and prolonging their survival time (Killian et al., 2016).

Although no technique is available yet to record the neural activities inside an organoid, 3D MEAs hold great potential in studying the organoid. Guihua et al. proposed a microneedle with five cellular-scale electrodes to detect glutamate concentration and cell activity in the brain simultaneously (Xiao et al., 2021). Five electrodes can be regarded as detecting one individual region of the brain because their positions are very close to each other. From the authors’ perspective, such a microneedle could also be inserted into the organoid to record both neural signals and glutamate concentration inside the brain. One new challenge would be how to insert the needle into the organoid without damaging cells. Micro-probe arrays are widely used *in vivo* to detect the cell activity of one particular region of the brain (Zhang et al., 2020). Motlagh et al. fabricated a high-density 3D pyramid-shaped MEA intended to record signals in our brain, as shown in Figure 7B (Ghane Motlagh et al., 2016a). In their follow-up study, polyethylene glycol, a biocompatible polymer, was coated on the surface of electrodes to improve the biocompatibility (Ghane-Motlagh et al., 2016b). If an organoid was cultured in these 3D MEAs and wrapped well around the needles, signals inside the 3D NoN could be tracked precisely and serve us better to understand more about the secrets of the human brain.

Organoids can also connect to each other through a robust fascicle consisting of axons (Kawada et al., 2017). It could be a platform for drug screening, even better than 2D NoN. Kirihara et al. proposed a microfluidic chip to build a cerebral tract model connecting two cortical regions by using hiPSC (Kirihara et al., 2019). The two spheroid chambers on the chip, each with a 2 mm diameter, were used for organoid culture, and they were connected by a microchannel. Axons could grow through the channel and connect two organoids, as shown in Figure 7C. Importantly, two organoids were electrically connected through the axon fascicle, which provides a platform for compound evaluation and drug screening.

## 5 Conclusion

In this study, we reviewed the latest designs in studying NoN and human brain models *ex vivo*. Multiple types of microsystems are discussed, including various microfluidic systems and MEAs. For better comparison, we summarized the representative nerve on chip models reported in prior art publications, including their advantage and limitations in Table 1. On the one hand, various neural models have been established on the basis of microfluidics owing to their parallel functions in controlling the microenvironment of cells and manipulating particles, including cells. On the other hand, various structures of MEAs have been designed for high-quality neural activity recordings to better understand neural interconnections inside the brain. Despite the fact that remarkable progress has been made in neuroscience in recent years, there are still many challenges that remain to be addressed. First, central hypoxia, the main reason for cell death in 3D cell culture, greatly limits research on organoids. In this respect, biomaterials that enable smooth nutrient transport and innovative cultivation are required. Second, co-culture of neural cells with other cells of interest is still hard to achieve due to different nutrient requirements per cell type. This greatly hinders the study of nerve–organ connections. Third, whether an artificial NoN can be trained to achieve desirable functions is still an unknown factor. Despite global efforts in recording the signals and stimulating the neurons of NoN on chip, none of these studies reported NoN systems obtained a simple real function. Some research studies have built biomimetic neural networks with computers, providing a new potential strategy to train the artificial NoN (Khoyratee et al., 2019).

**TABLE 1:** Comparison of the performance of representative nerve on chip models reported in prior art publications.

Type	Recording method	Biomaterials	Applications	Advantages	Limits	Ref
Lollipop-shaped nerve on chip	Patch clamp	iPSC derived from human neurons and primary human Schwann cells	Screen therapeutic molecules and study neuropathology	All human cells, similar to *in vivo* peripheral nerve	One nerve only, no connection between neurons	Sharma et al. (2019)
Concentration gradient chip	No recording	Fetal mouse cerebral cortex neurons	Study isolated axons in various soluble gradients	Stable solution gradient, good variable control	No detection during the growth of axons	Taylor et al. (2015)
Mobile neural microplates	No recording	PC12 cells	Precise neural circuits build	Free to design, single-cell manipulation	Limited growth of neurons	Yoshida et al. (2016)
Microtunnel device	Fluorescent intensity	Hippocampal neurons from rats	Study rapid neuroprotection	Novel recording method	Low-resolution detection	Samson et al. (2016b)
Three compartment MEA chip	96 Titanium nitride electrodes	Human embryonic stem cell derived human neurons	Focal epilepsy models for drug testing	Communications between different brain region were simulated	Unable to record intracellular potentials	Pelkonen et al. (2020)
Nanoelectrode array	4,096 Pt-black coated electrodes	Rat neurons from the cortex, hippocampus, and ventricular zones	Large-scale network of neurons mapping	Both exo-/intracellular signals can be recorded	Two-dimensional recording only, network of neurons has no function	Abbott et al. (2020a)
Long-term perfusion MEA chip	60 micro-electrodes	Brain slice	Long-term culture and record brain tissue *in vitro*	Long-term culture and perfusion	Low resolution and sensitivity to study network of neurons	Killian et al. (2016)
3D MEA chip	Microneedle electrodes	Neuroblast cell line (CCL-131)	Interface between bioelectronic devices and tissues	Three-dimensional electrodes	No connection between cells, only applicable to tissue	Ghane-Motlagh et al. (2016)

Presently, there are many directions we can work to further improve the NoN models *ex vivo*. First, there is still room to further improve the regular and long-term cell culture techniques. For example, neurons differentiated from hiPSC do not perform as good as primary neurons in our body. Differentiation protocols or devices that better mimic the microenvironment *in vivo* are required urgently to increase the differentiation efficiency and accuracy, which can both greatly reduce the sacrifice of rats and improve *ex vivo* brain models. Although some studies have achieved the culture of neurons on chip up to 1 year, neural activity gradually decreases after two or 3 months, indicating that cells are senescent. Second, materials with multiple functions, including high transparency, low impedance, and permeability, are welcome. Better flexibility in fabrication allows us to design amazing MEAs and microfluidics more freely. If allowed, central hypoxia could be addressed by inserting blood vessel-like new materials into organoids. Third, a rapid, high-resolution, and 3D fabrication method is needed for better building the chips for organoid culture. Also, a rapid, high-resolution 3D bioprinter technique is much needed to print elaborate brain-shaped scaffolds. In this case, neuron cells can be embedded on the scaffolds and survive for up to 10 years. Eventually, we may build an advanced artificial brain *ex vivo* and train it *via* current or various chemical stimulations. “brain in a vat” may not be a dream anymore.

The study of the neural system will eventually promote the medical treatment of neural diseases and profound investigation of the human brain. With the rapid development in neuroscience, neural diseases may have a chance to be cured in the future. In related subjects of neuroscience, such as bio-inspired robots, artificial intelligence can have a qualitative leap.
